# Sickle Cell Disease and Male Infertility: Pathophysiological Mechanisms, Clinical Manifestations, and Fertility Preservation Strategies—A Narrative Review

**DOI:** 10.3390/life16020192

**Published:** 2026-01-23

**Authors:** Christos Roidos, Aris Kaltsas, Evangelos N. Symeonidis, Vasileios Tzikoulis, Nikolaos Pantazis, Chara Tsiampali, Natalia Palapela, Athanasios Zachariou, Nikolaos Sofikitis, Fotios Dimitriadis

**Affiliations:** 1 First Department of Urology, Faculty of Medicine, School of Health Sciences, Aristotle University of Thessaloniki, 54124 Thessaloniki, Greece; drchriroid22@gmail.com (C.R.); bill1996tziko@gmail.com (V.T.); nikospant94@gmail.com (N.P.); 2 Third Department of Urology, Attikon University Hospital, School of Medicine, National and Kapodistrian University of Athens, 12462 Athens, Greece; ares-kaltsas@hotmail.com; 3 Department of Urology II, European Interbalkan Medical Center, 55535 Thessaloniki, Greece; evansimeonidis@gmail.com; 4 Independent Researcher, 55131 Thessaloniki, Greece; x.tsiampali@gmail.com; 5 Medical Faculty, Medical University of Sofia, 1431 Sofia, Bulgaria; nataliapalapela21@gmail.com; 6 Department of Urology, Faculty of Medicine, School of Health Sciences, University of Ioannina, 45110 Ioannina, Greece; zahariou@otenet.gr (A.Z.); nsofikit@uoi.gr (N.S.)

**Keywords:** sickle cell disease, male infertility, oxidative stress, fertility, hypogonadism, spermatogenesis

## Abstract

Sickle cell disease (SCD) is an inherited hemoglobinopathy in which hemoglobin S polymerization drives hemolysis and vaso-occlusion with progressive organ morbidity. Male reproductive impairment is increasingly recognized but remains underreported. This narrative review summarizes mechanistic pathways, clinical manifestations, and fertility preservation options relevant to men with SCD. PubMed, the Cochrane Library, and Medscape were searched through 31 December 2025 for human studies addressing endocrine changes, semen quality, priapism and erectile dysfunction, oxidative stress, and treatment-related gonadotoxicity. Evidence supports converging mechanisms: recurrent vaso-occlusion and chronic hypoxia may injure the seminiferous epithelium and impair Leydig cell steroidogenesis; oxidative stress and inflammation contribute to sperm DNA and membrane damage; and disease-modifying or curative therapies such as hydroxyurea and hematopoietic stem cell transplantation can further compromise spermatogenesis. Clinically, men with SCD may present with oligozoospermia, azoospermia, hypogonadism, and sexual dysfunction, particularly after recurrent ischemic priapism. Fertility preservation should be discussed early, ideally before prolonged hydroxyurea exposure or transplantation, and may include semen cryopreservation and testicular sperm extraction (TESE) with assisted reproduction when needed. Prospective longitudinal studies are required to define reproductive trajectories and optimize counseling and management.

## 1. Introduction

Sickle cell disease (SCD) comprises a group of autosomal recessive hemoglobinopathies caused by a point mutation in the beta-globin gene (HBB). The substitution of valine for glutamic acid at position 6 produces hemoglobin S (HbS); homozygosity for the HbS allele results in sickle cell anemia, the most severe clinical phenotype [1,2]. Since Herrick’s 1910 description of abnormally shaped erythrocytes, SCD has been recognized as highly prevalent in populations from sub-Saharan Africa and other regions historically affected by malaria, including parts of the Mediterranean basin, the Middle East, and India [3]. Under low-oxygen conditions, HbS polymerizes and promotes red-cell dehydration and rigidity, increasing blood viscosity and impairing microvascular flow. These biophysical changes drive chronic hemolysis and episodic vaso-occlusion, resulting in acute ischemic events and progressive organ damage over time [4]. Genitourinary involvement includes hematuria and papillary necrosis, recurrent urinary infections, and erectile dysfunction that is often linked to priapism [1].

Male reproductive abnormalities in SCD are clinically important yet frequently overlooked. Reported findings include delayed puberty, reduced testicular volume, and features consistent with androgen deficiency [5]. The pathogenesis appears heterogeneous and may reflect primary testicular injury from repeated microinfarction and chronic hypoxia, potential disruption of the hypothalamic–pituitary–gonadal axis, nutritional factors such as zinc deficiency, and constitutional growth delay [6,7,8]. At the molecular level, the factors linking SCD to male infertility can be conceptually grouped into vascular causes (ischemic injury from vaso-occlusion), endocrine disturbances (hypothalamic–pituitary–gonadal dysregulation and gonadal steroid impairment), oxidative stress (excess reactive oxygen species causing cellular and DNA damage), and genetic determinants (the HBB mutation and modifiers of disease severity) [9]. These mechanisms often overlap and collectively contribute to impaired spermatogenesis and sexual function in affected patients. Despite improving survival into reproductive age, guidance on fertility risk assessment and preservation in men with SCD remains fragmented. This review integrates endocrine, andrologic, and urologic evidence to outline the mechanisms that link SCD and its therapies to impaired spermatogenesis and sexual dysfunction, and to summarize practical fertility preservation strategies.

## 2. Methods: Narrative Review Approach

### 2.1. Literature Search (Narrative Review)

PubMed, the Cochrane Library, and Medscape were searched for English-language publications from database inception to 31 December 2025. Search terms were used alone and in combination and included: (“sickle cell disease” OR “sickle cell anemia”) AND (“male infertility” OR semen OR spermatogenesis OR hypogonadism OR testosterone OR “erectile dysfunction” OR priapism). Where relevant, the terms oxidative stress and hydroxyurea were added. Reference lists of eligible articles and relevant reviews were also screened to identify additional studies.

After duplicate removal, two reviewers independently screened titles and abstracts for relevance and assessed full texts against predefined eligibility criteria. Eligible studies reported human data on at least one male reproductive outcome in the context of SCD, including reproductive endocrine function, semen parameters, sexual dysfunction (priapism and/or erectile dysfunction), oxidative stress or redox biomarkers, and fertility preservation or assisted reproduction. Non-English publications, animal-only studies, and reports not addressing male reproductive outcomes in SCD were excluded. Key study characteristics, participant features, outcomes, and principal inclusion and exclusion criteria were extracted using a standardized form.

### 2.2. Study Selection, Data Extraction, and Approach to Study Quality

Data were extracted using a predefined template and cross-checked by a second reviewer to improve accuracy. When multiple publications appeared to derive from overlapping cohorts, the most informative or most recent report was prioritized. Given the narrative scope of the review and heterogeneity in study design, populations, and outcome definitions, a formal risk-of-bias tool was not applied uniformly across all study types; instead, methodological features (e.g., sample size, exposure ascertainment, laboratory methods, and confounding control) were considered qualitatively.

**Figure 1:**
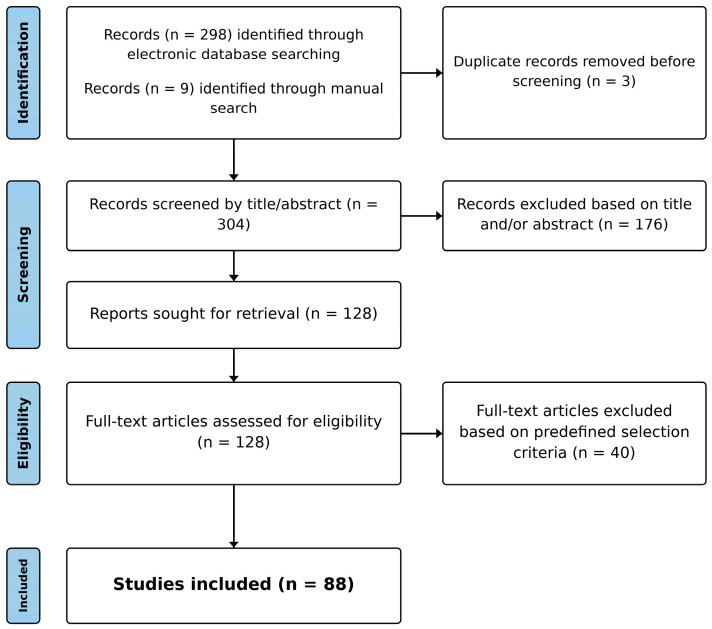
Literature identification and inclusion (narrative synthesis). The initial electronic search identified 304 records, of which 88 publications met the inclusion criteria and were included in the final narrative synthesis.

Evidence was synthesized narratively, emphasizing consistency of findings, study limitations, and clinical or translational relevance. Quantitative pooling was not performed because outcome measures, assays, and reporting were highly variable across studies. The electronic search retrieved 304 records; after screening, 88 publications were included in the final narrative synthesis (Figure 1).

## 3. Narrative Evidence Synthesis

### 3.1. Endocrine Phenotypes and Hypogonadism

Endocrine abnormalities are frequently reported in men with SCD and circulating testosterone concentrations are often lower than in healthy controls. Across cohorts, gonadotropin patterns vary, suggesting that both primary testicular injury and, in some patients, central dysregulation may contribute; disease severity and nutritional status may further modify these profiles [5,10,11,12,13,14,15]. Several studies describe low testosterone accompanied by elevated luteinizing hormone (LH) and/or follicle-stimulating hormone (FSH) and reduced markers of Sertoli cell function, which is consistent with combined Leydig and Sertoli cell impairment and disordered spermatogenesis. Chronic hypoxia, oxidative stress, and recurrent vaso-occlusive injury are plausible contributors to this phenotype [5,11,12].

Earlier endocrine surveys also interpreted some hormonal patterns as compatible with secondary hypogonadism. In one study, men with more severe disease tended to have lower LH, FSH, testosterone, and cortisol than those with milder phenotypes, with endocrine abnormalities clustering in the former group [10,11]. Conversely, other series have reported low testosterone with increased FSH and/or prolactin and without evidence of pituitary suppression, supporting a predominant testicular component in many patients [12]. Dynamic assessment of the hypothalamic–pituitary–testicular axis, combined gonadotropin-releasing hormone (GnRH) and thyrotropin-releasing hormone (TRH) stimulation (GnRH–TRH) in small cohorts generally demonstrates preserved pituitary responsiveness, arguing against a uniform hypothalamic–pituitary defect across SCD populations [15]. Similarly, reduced baseline androgens with an augmented gonadotropin response to GnRH have been interpreted as primary hypogonadism in adult men with SCD [5]. Clinically, reduced paternity rates have been described among affected men, whereas many women with SCD can achieve pregnancy, highlighting a sex-specific reproductive burden [16].

Recent endocrine cohorts highlight a mixed pattern that includes compensated hypogonadism and iron-related androgen suppression [17]. In a cross-sectional study of 80 adult men with SCD, higher ferritin was associated with lower testosterone and higher LH, supporting iron-overload-related gonadal dysfunction [18]. In a Brazilian cohort of adult men with SCD, compensated hypogonadism was frequent at 26.4%, suggesting that subclinical Leydig cell stress may be common and merits longitudinal monitoring [19].

### 3.2. Nutritional and Biochemical Contributors (Zinc and Related Factors)

Abbasi et al. (1976) [5] also evaluated zinc levels in individuals with SCD. They found that zinc levels were decreased, and there was a positive correlation between erythrocyte zinc and serum testosterone levels in these patients [5].

Zinc deficiency, often resulting from urinary losses, is common in SCD patients. Studies have suggested that zinc deficiency in adolescent males with SCD is associated with growth retardation and hypogonadism [20]. Zinc deficiency is known to adversely affect testicular function in both humans and animals. A review of the effects of a severe zinc-deficient diet on testicular function in rats revealed a significant reduction in the diameter of seminiferous tubules [21]. Additionally, serum testosterone levels were decreased, while serum FSH was elevated, and the rats were azoospermic [22]. These findings underscore the essential role of zinc in spermatogenesis and testosterone steroidogenesis. Zinc should, therefore, be considered a key factor contributing to impaired testicular function in male individuals with SCD [23].

To address zinc deficiency, one study recommended the use of oral zinc supplementation [24]. Although zinc supplementation in this population has been shown to improve testosterone levels and promote longitudinal growth, the precise mechanisms remain to be fully elucidated [25]. Based on these observations, the authors concluded that androgen deficiency in patients with sickle cell anemia can be effectively corrected with zinc supplementation.

### 3.3. Semen Profile and Sperm DNA/Chromatin Integrity

The mean life expectancy for individuals with SCD is currently around 50 years [26], and although their quality of life has significantly improved, many more patients are now surviving to reproductive age. Oligozoospermia and azoospermia are commonly observed in male patients with SCD [27]. Sperm analysis in these patients frequently reveals low sperm count and impaired motility of spermatozoa [28]. The similarities of these findings to those seen in men with varicocele led researchers to conclude that testicular hypoxia, possibly resulting from the chronic vaso-occlusion in SCD, is a common stressing factor.

A study conducted in Nigeria on 23 male SCD patients found no significant difference in ejaculate volume compared to controls, but considerable impairments were observed in sperm concentration, total sperm count, motility, and morphology [16]. The investigators noted that subfertility appears to be a more significant issue for males than females with SCD, as many women with the disease have had successful pregnancies, while male patients rarely father children. These results were similar to those from another study [29], which reported significantly reduced sperm motility, density, normal sperm morphology, and ejaculate volume in SCD patients. Additionally, there was an increase in the percentage of immature spermatogenic cells and abnormal spermatozoa, with amorphous and tapered heads. These findings highlight the association between abnormal semen parameters and SCD. The observed reduction in ejaculate volume warrants further investigation into potential abnormalities in the accessory sex organs, such as the seminal vesicles and prostate gland, which contribute up to 80% of ejaculate volume. Some studies also reported a decrease in sperm vitality [14].

In another study, 76 semen samples were collected from 34 patients before they began treatment with hydroxyurea (HU) [30]. The most affected sperm parameters were forward motility and morphology. In 40.3% of the samples, total sperm count was decreased, although no cases of azoospermia were observed. The volume of semen was normal in 74.3% of the samples. Notably, only three patients (9%) had normal sperm parameters across all measures. There was no correlation between mean baseline hemoglobin level or percentage of baseline fetal hemoglobin and sperm parameters. The same study revealed significant declines in sperm concentration, motility, and morphology in SCD patients compared to age-matched healthy controls [30]. The mean sperm concentration in the SCD group was 12.4 ± 3.2 million/mL, significantly lower than the 42.8 ± 5.6 million/mL observed in controls (*p* < 0.001). Progressive sperm motility was also compromised, with 21.3% ± 4.6% of sperm showing forward motility in SCD patients, compared to 58.7% ± 5.2% in healthy controls (*p* < 0.001). Morphological assessment revealed that more than 80% of spermatozoa in the SCD group exhibited abnormalities, predominantly affecting the head and midpiece, which is consistent with oxidative damage to sperm DNA and membranes [31].

A prospective study of 100 men with SCD undergoing serial semen assessments reported that hydroxyurea, even at low doses, was associated with incident oligozoospermia and azoospermia and that semen parameters normalized after a 3-month interruption in approximately 73% of affected patients [32]. In a multicenter comparative study of adolescents and young adults, semen abnormalities were common but did not differ significantly between those exposed to hydroxyurea before puberty and hydroxyurea-naive patients, although transfusion exposure differed substantially between groups [33]. A 2024 systematic review and meta-analysis found that hydroxyurea was associated with lower sperm concentration and total sperm count, and these reductions persisted after treatment cessation, whereas semen volume, initial forward motility, and morphology were not significantly affected [34]. Not all cohorts replicate severe impairment, and an Indian case–control study reported no evidence of low serum testosterone or poor semen quality in adult men with SCD, underscoring between-cohort variability [35].

### 3.4. Testicular Injury: Histopathology and Imaging

The lungs, spleen, kidneys, bone marrow, and brain are commonly affected by infarcts due to vaso-occlusion caused by sickle cells. While rare, SCD can also lead to testicular infarction. Li et al. (2003) described a case of a patient with SCD who experienced a testicular infarct [8]. Histological findings from this case were categorized into acute (several days old) and recent (2 to 3 weeks old) infarcts. The acute infarct showed necrotic seminiferous tubules associated with nuclear debris, congestion, and acute inflammatory infiltrates. In contrast, the recent infarct demonstrated coagulative necrosis without nuclear debris, no acute inflammatory response, and vascular proliferation at the periphery.

Additionally, remote infarctions were observed, evidenced by atrophic seminiferous tubules, fibrotic scarring, and hemosiderin deposition. The presence of maturation arrest and hypospermatogenesis could be attributed to such infarctions and/or chronic hypoxia resulting from vascular insufficiency and anemia [28]. Notably, these old infarcts were clinically silent, as the patient had not experienced any testicular pain prior to diagnosis. Based on these findings, Li et al. proposed that silent testicular infarctions contribute to testicular atrophy and failure.

Beyond histopathology, quantitative imaging suggests measurable testicular structural changes in SCD [36]. In a prospective shear wave elastography study, men with SCD had lower testicular volumes and higher stiffness values than controls, and 28% showed impaired sperm parameters; age and hydroxyurea use were associated with worse semen outcomes [36]. In a pediatric cross-sectional ultrasound study, testicular volume trajectories differed across puberty in boys with homozygous hemoglobin S (HbSS) versus controls, and the frequency of testicular pain episodes predicted volume changes, supporting a link between vaso-occlusive burden and gonadal development [37].

### 3.5. Sexual Dysfunction: Priapism and Erectile Dysfunction

Priapism, typically ischemic (low-flow), is a well-recognized urologic complication of SCD and a major driver of subsequent erectile dysfunction [1,38,39,40]. In cohort studies, roughly one-third of affected males report at least one episode, often beginning in adolescence, and erectile impairment becomes more likely with recurrent or prolonged attacks [38,39,41]. Physiologically, erection depends on cavernosal smooth muscle relaxation, increased arterial inflow, and restriction of venous outflow. In SCD, deoxygenation within the corpora promotes erythrocyte sickling, increases viscosity, and favors sinusoidal stasis. Endothelial activation, altered nitric oxide bioavailability, and prothrombotic signaling may further contribute, with proposed mechanisms including microvascular plugging, thrombosis, vasospasm, and intimal hyperplasia [42,43]. The resulting hypoxia and acidosis amplify sickling and can create a self-sustaining ischemic cycle.

When ischemia persists, inflammation and reperfusion injury can damage cavernosal smooth muscle and promote fibrosis of the trabecular framework, providing a mechanistic link between priapism duration and long-term erectile dysfunction [1,41]. Clinically, ischemic priapism presents with a painful, rigid corporal shaft with relative glans sparing, and episodes commonly occur during sleep when hypoventilation and acidosis lower oxygen tension. The pain reflects tissue ischemia; prolonged events require urgent management to limit irreversible structural injury [1]. Evaluation typically includes a complete blood count and basic chemistry profile. When the sickle status is unknown, hemoglobin electrophoresis is indicated. If the clinical phenotype is unclear, cavernosal aspiration with blood gas analysis can help distinguish ischemic from non-ischemic priapism; ischemic samples demonstrate low oxygen tension with hypercapnia and acidosis [44].

Although much attention has focused on nitric oxide and phosphodiesterase pathways, emerging work also implicates impaired vasoconstrictive signaling in SCD-associated priapism. The RhoA/Rho-kinase (ROCK) pathway normally supports cavernosal smooth muscle tone and the flaccid state [45,46]. In transgenic sickle-cell mouse models, downregulation of RhoA/ROCK signaling has been associated with exaggerated erectile responses and a priapic phenotype. In particular, reduced ROCK activity and decreased ROCK2 expression have been reported in penile tissue from sickle mice, supporting a role for diminished contractile signaling in the pathophysiology of priapism [47,48,49,50,51].

Patient-reported outcomes emphasize that priapism and sexual dysfunction remain under-recognized in routine care [52]. In a survey study, priapism was reported by 32.6% of men with SCD versus 2% of controls, and erectile dysfunction prevalence was more than twofold higher in the SCD group; nearly half of men with SCD-related priapism had never sought medical attention [52]. In a Danish nationwide cohort of hemolytic disorders, SCD was the only disorder associated with priapism and the incidence rate of first priapism in SCD was 432.8 per 100,000 person-years [53]. A regional cohort also linked priapism presentations with markers of hemolysis and higher rates of SCD complications such as acute chest syndrome, stroke, and pulmonary hypertension [54].

### 3.6. Treatment-Related Gonadotoxicity

Disease-modifying and curative strategies have improved survival and quality of life in SCD, bringing reproductive goals to the forefront. Common approaches include hydroxyurea, chronic transfusion programs, and hematopoietic stem cell transplantation (HSCT) [55].

Hydroxyurea (HU) is a ribonucleotide reductase inhibitor that increases fetal hemoglobin and reduces the frequency of painful vaso-occlusive crises. Because HU interferes with DNA synthesis, concerns persist regarding potential gonadotoxicity, particularly with prolonged exposure [56,57]. Preclinical data suggest that HU can worsen pre-existing SCD-related hypogonadism and adversely affect spermatogenic endpoints in transgenic sickle mouse models [58]. Human observational studies likewise report deterioration in semen quality during HU therapy, including reductions in sperm concentration, motility, and normal morphology. Severe oligospermia and azoospermia have been described in some long-term users, although partial recovery after discontinuation has also been reported [30,59,60]. In the largest available series, impairment of semen parameters was observed within the first months after HU initiation and then tended to stabilize, suggesting an early treatment effect that persists with ongoing exposure [30]. Experimental and clinical observations indicate that HU can disrupt germ-cell chromatin organization and increase apoptosis in early spermatogenic populations, while spermatogonial stem cells may be relatively spared, allowing repopulation in some individuals after treatment interruption [30]. Beyond conventional semen parameters, limited data suggest that HU exposure may be associated with increased sperm DNA damage; however, assays and thresholds vary and causality remains uncertain [30]. For men who wish to preserve fertility, counseling before long-term HU therapy is recommended, including consideration of semen cryopreservation when feasible. Periodic semen assessment can be used to monitor changes, and contraception is typically advised during HU exposure and for several months after discontinuation in sexually active men with a potentially fertile partner [60].

Chronic transfusion therapy, another disease-modifying approach, carries its own reproductive implications. Regular blood transfusions can lessen SCD complications such as stroke and vaso-occlusive crises; however, a notable side effect of long-term transfusion is iron overload. Excess iron deposition in endocrine organs (particularly the pituitary and testes) can lead to hormonal dysfunction and impaired spermatogenesis [13]. If iron overload is untreated, transfusion-dependent patients may develop hypogonadotropic hypogonadism due to pituitary iron deposition and direct testicular damage [25]. Indeed, endocrinopathies related to iron overload are well-documented in transfusion-dependent hemoglobinopathies, occurring more frequently in thalassemia major but also observed in heavily transfused SCD patients [13]. Effective chelation therapy is therefore critical to mitigate iron-induced gonadal injury. Interestingly, improving oxygen-carrying capacity via transfusion may have short-term benefits on reproductive parameters. In one study of young men with SCD on chronic transfusions, a single transfusion significantly increased hemoglobin levels and was associated with acute increases in serum testosterone as well as improvements in sperm count and motility one-week post-transfusion [61]. These findings highlight the complex physiology whereby relieving anemia and hypoxia can transiently enhance sperm production, even as cumulative transfusions pose long-term risks. Overall, chronic transfusion programs should be coupled with iron monitoring and chelation to balance disease control with preservation of endocrine and reproductive health.

HSCT, while potentially curative for SCD, poses one of the greatest risks to male fertility. The myeloablative conditioning regimens used for HSCT (typically including agents such as busulfan and cyclophosphamide) are highly gonadotoxic, often resulting in permanent impairment of spermatogenesis. In a report of young male SCD patients, approximately half of those who underwent HSCT were azoospermic on long-term follow-up [55]. Even when spermatogenesis survives or recovers, reduced fertility and testosterone deficiency are common after transplant due to cumulative germ cell loss and Leydig cell damage. There are rare cases of normal semen quality after HSCT in SCD, demonstrating that fertility can occasionally be preserved, especially if less intensive conditioning is used or if transplantation occurs at a younger age [62]. Nevertheless, the default assumption is that curative HSCT will significantly compromise fertility, and patients should be counseled accordingly. Sperm banking is strongly recommended for postpubertal males prior to HSCT whenever feasible, and experimental approaches such as testicular tissue cryopreservation may be considered for prepubertal boys [55]. Reduced intensity and non-myeloablative transplant protocols may be less acutely gonadotoxic, but their long-term impact on fertility is not yet well defined [62]. Thus, while HSCT offers the hope of a cure, it necessitates a frank discussion of fertility risks and preservation strategies as part of pre-transplant counseling.

In a retrospective cohort of children and adolescents undergoing alkylator-based HSCT for SCD, post-transplant LH and testosterone levels in males were generally normal for age, although semen-based fertility outcomes were not systematically reported [63].

### 3.7. Oxidative Stress and Redox Imbalance

Available evidence indicates that men with SCD experience a systemic pro-oxidant milieu that may extend to the reproductive tract. Studies report higher lipid peroxidation (often assessed by malondialdehyde, MDA) together with lower activity of antioxidant enzymes, consistent with an imbalance between reactive oxygen species generation and antioxidant defenses [4,9]. Because spermatozoa and testicular tissue are highly susceptible to oxidative injury, this redox disruption provides a plausible pathway linking SCD to impaired semen quality. Reduced antioxidant capacity has been described for superoxide dismutase (SOD) and glutathione peroxidase (GPx) in SCD cohorts, suggesting that endogenous protective mechanisms may be insufficient under chronic inflammatory and hypoxic stress [9]. Such depletion may facilitate membrane lipid peroxidation and mitochondrial dysfunction, processes closely associated with reduced sperm motility and viability in male infertility more broadly [31]. Markers of oxidative DNA injury, including 8-hydroxy-2′-deoxyguanosine (8-OHdG), have been associated with poor sperm motility and abnormal morphology in infertile men and may be relevant in SCD, where oxidative stress is sustained and recurrent [31]. Collectively, these observations support oxidative stress as a key contributor to sperm DNA fragmentation and compromised fertilizing potential in SCD-associated male infertility.

### 3.8. Fertility Preservation and Assisted Reproduction: Practical Pathways and Research Gaps

Given the prevalence of subfertility and, in some series, azoospermia among men with SCD, fertility preservation should be integrated into routine care, particularly before interventions with potential gonadotoxicity such as prolonged HU therapy or HSCT. Early counseling allows patients to align disease-control decisions with reproductive goals. Semen cryopreservation is the most established option when ejaculated sperm are available, although baseline semen quality is often reduced in SCD, which may limit post-thaw yield. Even so, cryopreservation can provide an opportunity for future use with assisted reproduction and should be discussed proactively when treatment escalation is anticipated [30]. When semen parameters are inadequate for natural conception, assisted reproductive technologies (ARTs) such as intracytoplasmic sperm injection (ICSI) and in vitro fertilization (IVF) may be considered. In men with severe oligospermia or azoospermia, surgical sperm retrieval techniques (e.g., testicular sperm extraction, TESE) can enable ART by providing viable sperm directly from the testes [64,65,66,67]. In practice, fertility preservation in SCD is most effective when offered before irreversible testicular injury develops. A structured pathway that includes early reproductive counseling, timely sperm banking, and access to ART and surgical retrieval when indicated may improve the likelihood of biological parenthood for affected men.

In the largest fertility-center cohort to date, semen cryopreservation was feasible in most men with SCD, yet severe baseline impairments were common, supporting early referral before cumulative gonadal injury and before initiation of potentially gonadotoxic therapy [68].

## 4. Discussion and Clinical Implications

**Figure 2:**
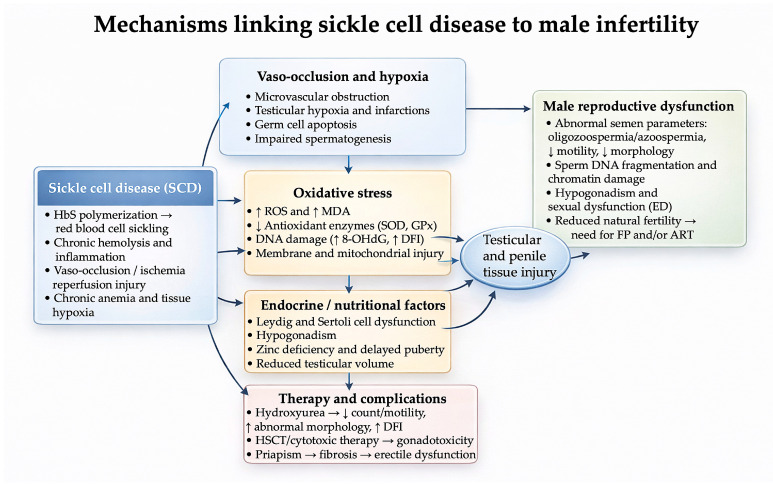
Proposed mechanisms by which sickle cell disease (SCD) may impair male fertility. Vaso-occlusion and chronic hypoxia, oxidative stress with impaired antioxidant defenses, endocrine and nutritional disturbances, and therapy- or complication-related gonadotoxicity converge on testicular and penile tissue injury, resulting in abnormal semen parameters, sperm DNA/chromatin damage, hypogonadism, erectile dysfunction, and reduced natural fertility with potential need for fertility preservation and assisted reproductive technologies. *Abbreviations:* ART, assisted reproductive technology; DFI, DNA fragmentation index; DHT, dihydrotestosterone; ED, erectile dysfunction; FP, fertility preservation; GPx, glutathione peroxidase; HbS, sickle hemoglobin; HSCT, hematopoietic stem cell transplantation; LH, luteinizing hormone; FSH, follicle-stimulating hormone; MDA, malondialdehyde; ROS, reactive oxygen species; RBC, red blood cell; SCD, sickle cell disease; SOD, superoxide dismutase; 8-OHdG, 8-hydroxy-2′-deoxyguanosine.

Male infertility in SCD appears to arise from interacting vascular, endocrine, and redox pathways that collectively impair testicular function and sperm competence (Figure 2).

Recurrent vaso-occlusive injury and chronic hypoxia can damage both the seminiferous epithelium and steroidogenic capacity, while oxidative stress and inflammation may further amplify cellular and DNA injury. Oxidative stress is a particularly compelling downstream pathway because it can integrate hemolysis-related inflammation, ischemia–reperfusion injury, and mitochondrial dysfunction. Across studies, oxidative imbalance correlates with poorer semen parameters and higher sperm DNA fragmentation, and chronic hypoxia may perpetuate this redox disruption within the testes [69,70,71,72].

Microvascular damage and testicular ischemia may contribute to gonadal injury in SCD, as reduced red blood cell deformability promotes microvascular occlusion and impaired tissue perfusion [73]. Red blood cell-mediated microcapillary occlusion can be quantified using standardized microfluidic assays, with occlusion indices associated with clinical phenotype and hydroxyurea responsiveness [74]. Testicular ischemia has been documented clinically, with case reports describing absent intratesticular blood flow on Doppler ultrasonography and confirmatory ischemic necrosis in men with SCD [75]. Conservative management of segmental testicular infarction secondary to sickle cell anemia has also been reported, reinforcing ischemia as a plausible contributor to parenchymal loss [76]. Consistent with chronic ischemic remodeling, shear-wave elastography has shown increased testicular stiffness and reduced testicular volume in men with SCD compared with controls, and stiffness metrics have been associated with impaired semen parameters in a subset [36]. Beyond direct DNA fragmentation, oxidative stress can disrupt sperm epigenetic reprogramming, affecting DNA methylation dynamics and chromatin remodeling events relevant to embryo development [77].

Priapism warrants special emphasis because it can impose irreversible penile injury early in life. Prolonged ischemic episodes promote hypoxia and, upon reperfusion, inflammatory and oxidative cascades that culminate in corporal fibrosis and endothelial dysfunction. This trajectory provides a plausible explanation for the high burden of erectile dysfunction reported after recurrent priapism in SCD, with downstream consequences for sexual health and fertility planning [69].

HU has transformed SCD care, yet its reproductive safety profile remains a frequent patient concern. The current evidence base is dominated by observational studies and small cohorts, but it consistently signals declines in semen parameters during therapy, particularly in the first months. Reported reversibility after discontinuation is variable, emphasizing the need for individualized counseling and proactive fertility preservation before long-term exposure [30,59,60]. From a translational perspective, strategies that reduce hypoxia and oxidative injury within the testes may offer benefit beyond symptomatic management. Optimizing disease control, preventing recurrent vaso-occlusive events, and investigating targeted antioxidant or microcirculatory therapies are rational directions, but robust clinical trials in this area remain scarce.

In addition to disease-intrinsic pathways, external environmental factors may modulate SCD severity and further impact male reproductive health. Emerging data suggest that air pollution, particularly traffic-related ambient pollutants, can act as a trigger for acute SCD vaso-occlusive exacerbations, presumably via oxidative stress-mediated mechanisms [78]. Epidemiologic studies in urban settings have linked higher daily levels of pollutants such as carbon monoxide with increased emergency department visits for SCD pain crises, especially among children [79]. Such environmental stressors could exacerbate the hypoxic and inflammatory burden on the testes during vaso-occlusive episodes. Moreover, chronic exposure to environmental pollutants has been associated with direct effects on male fertility. For instance, men residing in highly polluted areas have been found to exhibit altered sperm nuclear protein composition and epigenetic profiles compared to those in cleaner environments [80]. In a recent molecular analysis, exposure to elevated air pollution corresponded with a lower protamine-to-histone ratio in sperm (indicating incomplete chromatin maturation and reduced DNA protection), as well as dysregulation of dozens of sperm microRNAs involved in oxidative stress responses and spermatogenesis [80]. These pollution-driven changes—impaired sperm chromatin packaging and miRNA expression—were not detectable by routine semen analysis but could plausibly contribute to reduced fertility or transgenerational effects [80]. Although such environmental influences on SCD-related infertility require further study, these findings underscore the importance of considering lifestyle and exposure factors in the comprehensive management of male patients. In addition, mechanistic evidence indicates that specific toxicants, such as hexavalent chromium, can interfere with sperm nuclear basic protein–DNA interactions, providing a plausible molecular basis for pollutant-associated disruption of sperm chromatin organization [81].

Mechanistic studies indicate that hemoglobin polymerization inhibition can improve hemorheologic properties of sickle blood by maintaining red blood cell deformability and reducing viscosity under deoxygenated conditions, and randomized trials of voxelotor demonstrate increased hemoglobin concentrations with reduced markers of hemolysis [82,83]. Ektacytometry-based assessments in voxelotor-treated cohorts further support improvements in red blood cell deformability and reduced sickling under deoxygenation [84].

Taken together, recent human studies support a pragmatic monitoring approach that couples endocrine screening with proactive fertility preservation [68]. Iron overload and compensated hypogonadism appear to be recurrent endocrine phenotypes, suggesting value in periodic testosterone, gonadotropin, and ferritin assessment within SCD follow-up [18]. Noninvasive testicular imaging, such as shear wave elastography, may help identify early parenchymal changes and stratify patients for closer reproductive surveillance [36]. Patient-reported data indicate that priapism-related sexual morbidity is common and frequently unmanaged, underscoring the need for standardized counseling and early urologic referral [52]. In transfusion-dependent thalassemia, magnetic resonance imaging (MRI)-derived anterior pituitary iron and gland shrinkage have been linked to declining LH/FSH concentrations and impaired reproductive status, providing a surveillance framework potentially relevant to heavily transfused men with SCD [85].

Clinical endocrine phenotyping in SCD encompasses secondary and compensated hypogonadism, suggesting that fertility risk may reflect mixed central and testicular mechanisms across disease phenotypes [19]. Contemporary reviews estimate hypogonadism prevalence in SCD males up to approximately 25% and highlight reproductive health implications of testosterone deficiency [69]. Because chronic opioid analgesia is common in SCD, opioid-induced androgen deficiency should be considered as a potentially reversible contributor to low testosterone [86]. In a controlled human cohort, opiate use was associated with impaired semen parameters and increased sperm DNA damage, highlighting analgesic exposure as a potential confounder when interpreting semen and endocrine phenotypes [87]. Recent expert commentaries emphasize integrating fertility, contraception, sexual health, and genetic counseling into routine comprehensive SCD care for adolescents and young adults, particularly as disease-modifying therapies expand [88].

## 5. Limitations of the Evidence and Research Priorities

The current evidence linking SCD to male reproductive dysfunction is dominated by small, single-center, cross-sectional or retrospective studies, which limits causal inference and generalizability across health systems and disease phenotypes [9,27]. Across reports, substantial heterogeneity in age, SCD genotype, disease severity, and comorbid exposures reduces comparability and increases residual confounding, particularly when men receiving hydroxyurea or chronic transfusions are analyzed alongside treatment-naïve cohorts [27,34]. Even within semen-focused studies, laboratory methods, reference standards, abstinence intervals, and reporting of morphology and motility are inconsistent, complicating synthesis and limiting the precision of pooled estimates [27,34].

Most studies emphasize conventional semen parameters and single-time-point hormones, whereas clinically informative outcomes such as paternity, time-to-pregnancy, ART utilization, and live-birth rates are rarely captured in a standardized manner [27,62]. Mechanistic inference in humans is further constrained by sparse use of reproductive biomarkers beyond gonadotropins and testosterone and by the limited integration of testicular imaging or histologic correlates in representative cohorts [27,36].

Therapy-related gonadotoxicity remains an area where evidence is suggestive but not definitive because exposure histories, dosing, adherence, and duration are variably documented and baseline fertility status is often unknown [27,34]. For hydroxyurea, observational data indicate deterioration in sperm parameters and uncertain reversibility in a subset, yet prospective, adequately powered longitudinal designs with harmonized semen and DNA integrity endpoints remain limited [30,34]. Data on hydroxyurea exposure before puberty are particularly constrained by small sample sizes and by the inherent inability to bank sperm prepubertally, leaving a critical gap in counseling for early initiation [33].

For curative approaches, long-term fertility outcomes after HSCT or emerging gene-based therapies are incompletely characterized, and conditioning-regimen heterogeneity makes it difficult to estimate absolute risks for individual patients [55,62]. Sexual dysfunction and priapism are frequently assessed using self-report instruments and retrospective recall, and many studies do not link priapism burden, erectile outcomes, and fertility planning within unified prospective frameworks [52,53]. Endocrine phenotyping is also inconsistent, with mixed patterns including compensated hypogonadism and iron-associated androgen suppression, but few studies include repeated measures to define trajectories or thresholds that predict infertility-related outcomes [18,19].

Research priorities should therefore center on multicenter prospective cohorts that follow boys through adulthood with standardized semen analysis protocols, repeat reproductive hormone panels, and prespecified clinical fertility endpoints including ART and live-birth outcomes [27,62]. Such cohorts should stratify by genotype and treatment exposure and incorporate objective measures of gonadal injury such as testicular volume and stiffness metrics, enabling earlier identification of men at highest risk and better timing of fertility preservation discussions [36,37]. Dedicated registries embedded in SCD programs could harmonize real-world data on hydroxyurea dosing, transfusion and iron metrics, priapism burden, endocrine status, semen trajectories, and fertility outcomes to support evidence-based counseling [18,27]. Priority gaps also include comparative reproductive safety studies of newer disease-modifying agents and curative platforms, using uniform reproductive endpoints and sufficiently long follow-up to detect delayed recovery or persistent impairment [62].

Finally, interventional studies should evaluate pragmatic strategies that may mitigate risk or improve counseling pathways, including optimized endocrine assessment, structured fertility preservation referral, and targeted correction of nutritional deficiencies where evidence suggests benefit, while avoiding untested empiric supplementation [20,24,69].

## 6. Conclusions

SCD is associated with clinically relevant male reproductive dysfunction arising from convergent vaso-occlusive, endocrine, and redox pathways that impair testicular function and sperm competence. Human evidence supports frequent abnormalities in semen parameters, mixed hypogonadal phenotypes, and substantial sexual morbidity, particularly after recurrent ischemic priapism.

Therapy-related effects should be incorporated into counseling and shared decision-making. Hydroxyurea is repeatedly linked to deterioration in semen quality in observational studies, with inconsistent recovery after discontinuation, supporting early discussion of semen cryopreservation when feasible. Chronic transfusion programs may alleviate hypoxia yet increase the risk of iron overload and endocrine disruption, underscoring the need for ferritin surveillance and appropriate chelation. Curative transplantation can be highly gonadotoxic, making pre-treatment fertility preservation essential.

A pragmatic clinical approach integrates reproductive history, targeted endocrine screening, semen analysis when appropriate, and early urologic referral for priapism and erectile dysfunction, with timely access to fertility preservation and assisted reproductive technologies. Key research priorities include prospective multicenter cohorts with standardized reproductive endpoints and comparative fertility safety data for newer disease-modifying and curative therapies.

## Data Availability

The original contributions presented in this study are included in the article. Further inquiries can be directed to the corresponding author.
